# Comment on “Propensity score-matched analysis comparing drains and no-drains in rectal cancer surgery: the value of using a hemostatic agent instead”

**DOI:** 10.1097/JS9.0000000000003119

**Published:** 2025-08-05

**Authors:** Ya-Hui Sun, Wei-Rong Cai, Ke-Jian Zou

**Affiliations:** a Department of Gastrointestinal surgery, Hainan General Hospital (Hainan Affiliated Hospital of Hainan Medical University), Haikou, Hainan Province, China; b Operating Room, Hainan General Hospital (Hainan Affiliated Hospital of Hainan Medical University), Haikou, Hainan Province, China

*Dear Editor,*

We read with great interest the article titled *“Propensity Score-Matched Analysis Comparing Drains and No-Drains in Rectal Cancer Surgery: The Value of Using a Hemostatic Agent Instead*^[1]^*.”* This study provides valuable insights into the use of Microporous Polysaccharide Hemospheres (MPH) as an alternative to prophylactic drains in rectal cancer surgery, and we commend the authors for their contribution to improving postoperative care in colorectal surgery. We confirm that this manuscript was prepared in accordance with the TITAN Guidelines 2025 for governing declaration and use of artificial intelligence in scholarly publishing^[2]^, and no AI tools were used in this work.

However, we would like to highlight a concern that could affect the robustness and generalizability of the findings. While the study considers important factors such as age, ASA classification, and comorbidities, the authors did not include the body mass index (BMI) of patients as a stratification factor in their analysis. BMI is a known determinant of postoperative recovery and complication rates in colorectal surgery^[3,4]^. Obese patients often face an increased risk of complications such as postoperative ileus, anastomotic leak, which could have significantly impacted the outcomes in the MPH and no-drain groups.

Excluding BMI as a stratification factor may overlook an important source of variability in postoperative complications and treatment responses. We respectfully suggest that future studies incorporate BMI as a key stratification factor to enhance the precision of the findings and better understand its role in the outcomes observed.

## Data Availability

The letter does not have any data.

## Research registration unique identifying number (UIN)

Not registered for the type of the study.
